# Therapeutical Notes

**Published:** 1872-12

**Authors:** Thomas A. Elder

**Affiliations:** Mifflintown, Pa.


ARTICLE VI.—*Therapeutical Notes.* By THOMAS A. ELDER, M.D., Mifflintown, Pa.

Sitting in my office this evening, looking over the JOURNAL—ever pleasant and profitable entertainment—for 1870, I noticed a "selection" upon the use of the sulphites in the treatment of tonsilitis, which I had read with a great deal of interest on its first appearance in the JOURNAL. And it occurred to me to say to your readers that my experience with the

SULPHITES IN TONSILITIS, agrees entirely with that of the writer of the above named selection. During the last two years tonsilitis has prevailed here very extensively, almost everybody being affected with it. Its symptoms it is unnecessary to record, being familiar to all the readers of Flint's Practice. Suffice it to say that it is ushered in by general languor, aching of back and limbs, headache, congestion and tumefaction of tonsils, with congestion of the half-arches and fauces, and fever; and the temperature in many cases is high, especially in children, ranging from 103° to 106½°, as tested by the thermometer in numerous cases.

I have not met with a single case which the sulphites, administered in sufficiently large and often repeated doses, would not promptly relieve and cure. The doses which I use are those recommended by Dr. Tyrrell, gr. xx to xxx, repeated every hour, for an adult, and correspondingly large doses for children. The fever is generally dissipated in twelve hours—rarely continues twenty-four. The soreness of throat, headache, etc., are generally as promptly relieved, and forty-eight hours are sufficient for a cure. In children, when saturated, they have produced sweating, and the peculiar cadaveric hue of sulphurous acid fumes.

When the disease has progressed to the stage of exudation—when the shoe-peg points begin to appear, or later—I have never met with a case which I thought was benefited by the sulphites. I am then accustomed to rely upon a prescription of Prof. Miller, for diphtheria, which has almost invariably given prompt and permanent relief.

| | | |
|---|---|---|
| "R. | Morphia Muriat., - - - - - | gr. ij. |
| | Acid, Muriat., dil., } Tr. Ferri Chloridi, } - - - - | aa dr. ij. |
| | Syrupi, - - - - - - - | oz. ss. |
| | Aquæ destillat., - - - - - - | oz. ij. |

M. Sig. Dose: A teaspoonful three or four times a day after water."

(I quote from memory.) The above to be modified according to circumstances.

As to the use of the

SULPHITES IN SCARLATINA,

I have had but two cases, which I think were undoubtedly scarlet fever, in which I had an opportunity of exhibiting them in the first stage.

The first case was that of Beckie P., æt. about 9 months, whose elder sister was and had been suffering from a very severe attack of scarlatina, in which suppuration of both tympani took place, with discharge on one side through the mastoid process, and in which total loss of hearing resulted for a time, followed by entire restoration. Little Beckie was a lively, playful, good humored child, and had free access to the room in which her sister lay sick. One morning her mother noticed that, instead of her usual life, activity, and frolic, she lay in her cradle with great disinclination to be disturbed, and some fever, but thinking it a mere "brash," did not call my attention to her. She remained in this condition until I called in the evening, when I found the following symptoms: skin, hot and dry; pulse, 140, full and bounding; throat, congested and swollen; temperature, 106½°; no rash. I ordered her gr. v of bi-sulphite of soda, every hour, in a tablespoonful of water. The next morning her temperature was 100½°. She had no more fever. I continued the remedy for forty-eight hours. She never had a symptom afterwards.

The second case was that of Mary W., æt. 14 months, a pale, delicate, but lively child, who had suffered from occasional attacks of intermittent fever through the summer. Her mother took her to bed apparently as well as usual. About eleven o'clock she felt of the child and found she was very warm, and on striking a light, discovered that she was very red all over. Not getting better, at one o'clock they sent for me. On arriving I found the child sleeping, but with occasional muscular twitchings; the skin hot, swollen, a perfect scarlet from head to foot, and apparently tender to the touch; throat swollen and inflamed. While making the examination she went off into a convulsion, which lasted three or four minutes. I ordered ice to the neck, and gave soda bi-sulphite, gr. vij, every hour. By morning the redness had left the skin, when a distinct rash, not very bright, was visible. The fever had somewhat abated, and at 5 o'clock P. M. she was entirely free, and had no return. The treatment was continued for forty-eight hours, when the rash had entirely disappeared. There were no further symptoms at that time. About two weeks afterward the sub-lingual, afterward the right sub-maxillary, and finally the right parotid gland became swollen and inflamed, with the formation and discharge of two small abscesses over the sub-maxillary. This inflammation has continued until the present time, now some six weeks, but it is subsiding.

If I am right in my diagnosis of these two cases, then I think we have a sure abortive of that dread scourge of child-life, scarlatina. I use the sulphite and bi-sulphite of soda, preferably the latter, on account of its not acting upon the bowels. I believe the main reason why they have been found inefficacious in many hands, is, that they have not been used aright. Let your readers take a "new departure" in their use, and I feel assured that, unlike our beloved democracy, they will not come to grief.

Again, in the same volume of the JOURNAL, I find an article on

BROMIDE OF POTASSIUM IN LEUCORRHŒA, by Dr. A. H. Kinnear, of Metamora, Ill., and it reminds me of a case I had a few weeks ago.

Mrs. S., æt. about 38 years, of nervo-sanguineous temperament, nursing her first live child, now 10 months old, called me in on account of sleeplessness in herself and child. I was unable to discover any cause for it, except it might be in her natural nervous temperament, heightened by her "new relations." I prescribed potass. brom., gr. x, before each meal, and on going to bed. In four days she slept well. There was also improvement in the babe's sleeping. About two weeks afterward she called me in again, and very privately, quietly, and confidentially (I am a young unmarried man, and the former had been my first visit to the house) told me she had such a "terrible flow of whites," especially at night, when she had been on her feet much during the day. It troubled her a great deal, so that she had to get up several times during the night to clean herself, so as to have any comfort. Had always been a hale, hearty woman, always regular, but had aborted at various stages of pregnancy, six times, but had not suffered any evil effects therefrom. Had never had "whites" before the birth of her child, but had had it ever since. Remembering the cases of Dr. Kinnear, I concluded to try the effects of the bromide, so ordered gr. xv, three times a day, and with the most happy effects, for in two weeks time she was entirely free from it, and quite happy.

As a sequel to the above case, I might relate that shortly afterward I prescribed the bromide in another case, but of just the opposite temperament, and rather poorly nourished, but it is needless to say, without any benefit, rather the reverse. Tr. ferri, gtts. x, and tr. nucis vom., gtts. v to gtts. x, three times a day, did much better.

Moral.—As everywhere else in medicine, so here, the multiplication table will not work.

AMMONIA IN SNAKE-BITE.

On the morning of Aug. 6th, 1872, Wm. R., Jr., colored, in getting out of his bed, which he occupied in common with his father and *their* wife, in their little hut at the foot of the mountain, was bitten by a snake—a rattlesnake or copperhead—was not certain which, on the back of the hand between the thumb and first finger. He sent Alice to town, four miles, for me, but not finding me, she returned, taking with her a pint of whisky, which they drank. The whisky having no effect on William, though it had on Alice, and the hand and arm swelling rapidly, and becoming very painful, he concluded to come to town and see me himself. He arrived about 5 o'clock P. M. It was with difficulty that he could get up stairs to my office. His hand and arm presented such a sight as I hope I shall not soon see again. They were swollen to such a degree that one would suppose the skin must burst; the skin seemed thick and hard like heavy leather; the pulse was small, weak, rapid, about 130, and very irregular; the breathing labored; the arm very painful; he was weak and faint. I injected with the hypodermic syringe, m. xv, of a solution composed of equal parts of aq. ammon and aquæ puræ, into the neighborhood of the bite, and m. x into the well arm. Within twenty minutes it showed its effect. He could feel it going all through him, especially at the point of injection. The pulse came down to about 80, doubled in strength, and became regular. Within an hour, the pulse growing somewhat more rapid, and weaker, I injected m. x more into the well arm, and at the same time, the same quantity into the affected arm. I ordered him spts. ammon. arom., dr. j, in water, every third hour. He then started on his homeward journey, and succeeded in going a mile, when he had to lie by for the night, being too weak to go farther. The next morning I saw him at the house in which he had spent the night. I found him in very much the same condition in which he was when he came to me the day before. The arm had become painful again, the pulse rapid and weak. I injected him again twice, in all about m. xx, of the above solution, and with the same result. On the evening of that day he succeeded in getting home, having walked all the way. The next day, toward evening, I saw him again. He had suffered but little pain, the pulse was regular, full and steady, the arm was diminishing in size, the appetite, which I neglected to mention was almost *nil*, improved. I gave him a brisk cathartic, and ordered him to continue the aromatic spirits of ammonia in half teaspoonful doses every third hour. In ten days the arm had regained its usual dimensions, some stiffness and soreness of the muscles being all that was left to remind him how near he had been to death. And being unable to work, he, and *their* wife departed for a more sunny clime, even "My Maryland," whence returning for winter quarters, I hope I may receive the reward of my services, or he be bitten by another "copperhead."

And now a word in regard to

CHLORAL HYDRATE, and I have done.

On Oct. 20th, 1872, Sabbath evening, I was called in to see J. M. B., a young man less than 21 years of age, of very fine mind, and of superior mental attainments, who was suffering with delirium tremens. He had been drinking more or less all through his academy and college course, and it finally culminated in this attack. He had only given manifestations of the delirium but a short time before I was sent for. He was rather quiet, but the illusions were quite distinctly marked. He would say very quietly "there they are," but occasionally crying out in terror, and turning his back, would strike out with his hands at the demons that were approaching him. His eyes were staring, the pupils very widely dilated, the whole eye protruding, and he could not wink; the pulse was rapid, strong and bounding. I gave him hydrate chloral, gr. xxij, in equal parts of syr. tolu, and aq. menth. vir. About twenty minutes after he had taken it he could move his eyelids a little, the pulse was somewhat slower and weaker. A half hour after the first dose, gave him another: still more improvement; could wink; the demons had departed, never more to return. In three-quarters of an hour gave him another dose, and repeated it twice more, and at midnight he was put to bed, when he slept nearly all the remainder of the night. Next morning he was of course somewhat nervous and weak, and very solicitous to know whether "they" would come back. I assured him that they would not. I prescribed potass. brom., gr. xv, every third hour through the day, and the next morning he was able to go away with his father, who on learning of his condition, had come to take him home. In this case the chloral acted like a charm.

